# A case of crossed-doubled patellar tendon: an atavistic variant, simple mutation or pathologic finding?

**DOI:** 10.1007/s00276-016-1706-x

**Published:** 2016-06-15

**Authors:** Alexander Loizides, Carmelo Messina, Bernhard Glodny, Leonhard Gruber, Erich Brenner, Hannes Gruber, Benjamin Henninger

**Affiliations:** 1 Department of Radiology, Medical University Innsbruck, Anichstrasse 35, 6020 Innsbruck, Austria; 2 Postgraduate School in Radiodiagnostics, Universita degli Studi di Milano, Milan, Italy; 3 Department of Clinical and Functional Anatomy, Medical University Innsbruck, Innsbruck, Austria

**Keywords:** Patellar tendon, Knee, Anatomical variant, Doubled-crossed

## Abstract

Anatomical variants can be found throughout the whole body. Especially in the knee region, some variability has been reported concerning the osseous, tendinous, and muscular system. Beside a few cases of patellar tendon aplasia, no anatomical variations of this tendon are known. We present a rare case of a doubled patellar tendon as an anatomical variant, which to our knowledge, has not been described previously.

## Introduction

The patellar tendon (PT) forms a part of the knee extensor mechanism and acts as a passive stabilizer of the patella, a sesamoid bone embedded within the substance of the quadriceps tendon (QT) [2]. The PT consists of fibrous connective tissue that is composed of densely packed collagen fibre bundles aligned parallel to the longitudinal tendon axis surrounded by a tendon sheath, which also consists of extracellular matrix (ECM) components. Its fascicles originate from the distal two-thirds of the anterior surface of the patella. The origin has usually a crescent shape aspect, with the medial and lateral fascicles attached to the patella more proximal than the central fascicles [2]. Along its course, the PT is usually oriented parallel to the long axis of the proximal tibia and fans out to insert on the tibial tuberosity, distally mingling with the fascial expansions of the iliotibial tract [1].

Several tendons and ligaments are found in the knee, many of which may present anatomical variants. We present a rare case of a doubled patellar tendon as an anatomical variant, which to our knowledge, has not been described previously.

## Case description

A 30-year-old female office administrator was admitted with a 6-month history of increasing left knee pain. In 1990 and 1995, she underwent surgery for removal of a haemangioma located near the medial border of the patella. Examination of the knee showed no abnormalities, and angulation was possible without any significant limitation in the range of motion. On physical examination, she had spontaneous pain in the anterior region of the knee, with a maximum point of tenderness just near the surgical scar, at the superomedial corner of the patella. In addition, pressure on the patella itself was painful. Magnetic resonance imaging (MRI) was performed for further evaluation (3T Skyra, Siemens, Erlangen, Germany). The protocol consisted of T1- and T2-weighted images as well as fluid-sensitive short tau inversion recovery (STIR) sequences and contrast enhanced T1-weighted images. MRI revealed two different angiomatous masses that were the most likely cause of the patient’s pain. The bigger one with a maximum extension of almost 7 cm and a fusiform shape with partial intraosseous extension were located on the medial edge of femoral diaphysis and arising from the upper part of the medial aspect of the intercondyloid fossa. The smaller mass was located on the medial upper border of the patella, with a maximum diameter of 2 cm. Both of these lesions were diagnosed as hemangiomas.

**Fig. 1:**
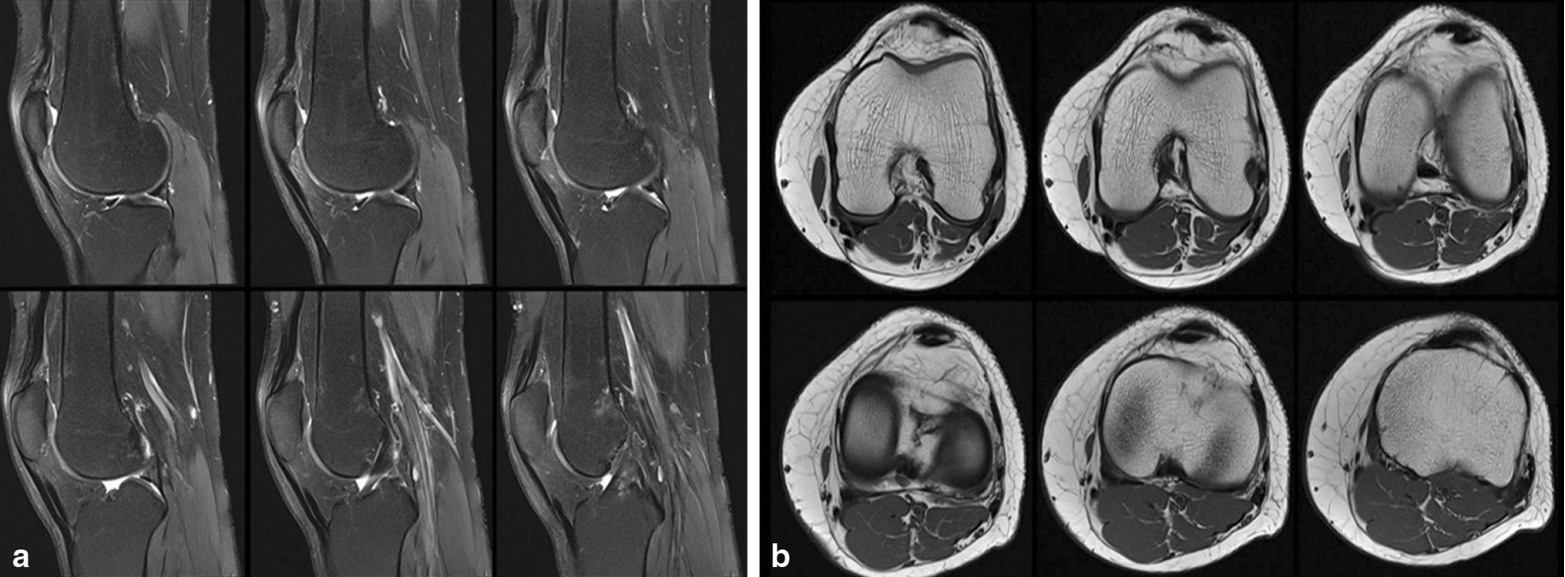
Sagittal intermediate-weighted fat-suppressed images (TE 38 ms; TR 3950 ms; matrix 448 × 448, slice thickness 3 mm) of the right knee in six different slice positions (**a**). A bifid, low-signal intensity, band-like structure, separated by a thin fat strip along the whole course of the PT can be shown on each image. Axial T1-weighted images (TE 19 ms; TR 626 ms; matrix 448 × 378, slice thickness 3 mm) of the right knee in six different slice positions starting at the height of the femur condyles (*first row*, *first image*) and running distally until the tuberosity of the tibia (*second row*, *third image*), and depicting the two crossing bundles of the patellar tendon (**b**)

**Fig. 2:**
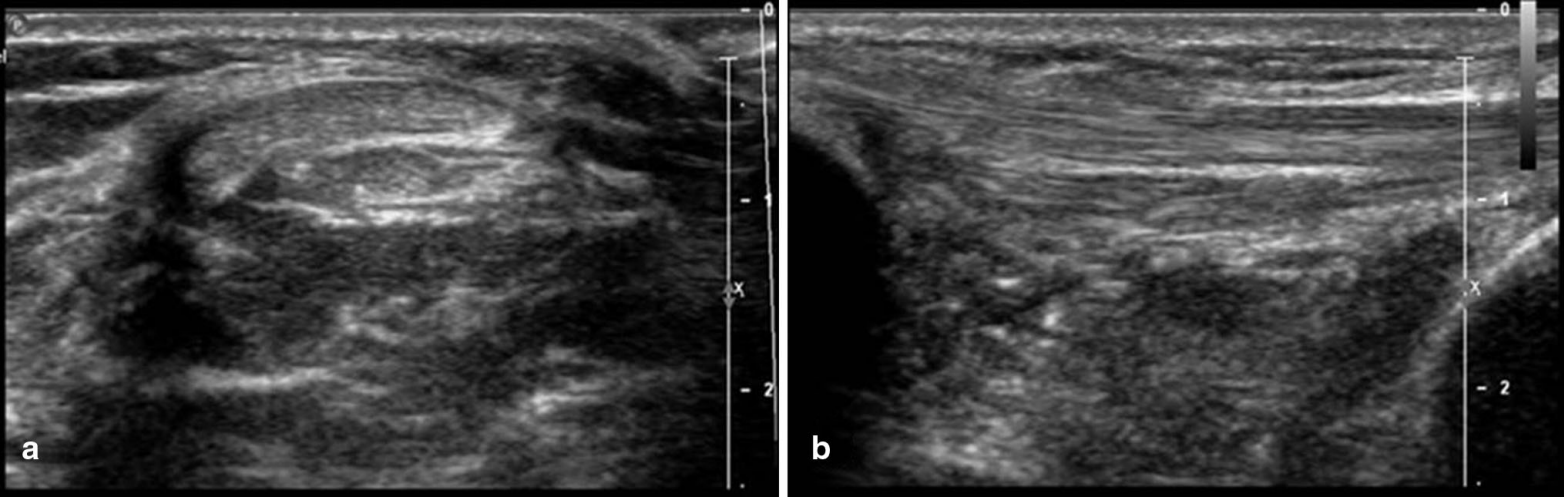
Grey-scale transverse (**a**) and longitudinal (**b**) scan depicting the two bundles of the patellar tendon

As an additional finding, a PT composed of two distinct bundles originating from the anterior surface of the inferior pole of the patella, with a double insertion on the tibial tuberosity was noticed. These two tendon bundles were separated by a thin fat strip along their whole course and crossed each other: the medially originating bundle inserted on the typical position of the tibial tuberosity, whereas the lateral one inserted on the medial aspect of the tibial tuberosity, almost in continuity with the ipsilateral patellar retinaculum (Fig. 1). The doubled PT was additionally confirmed on a subsequent grey-scale sonography (Fig. 2).

## Discussion

**Fig. 3:**
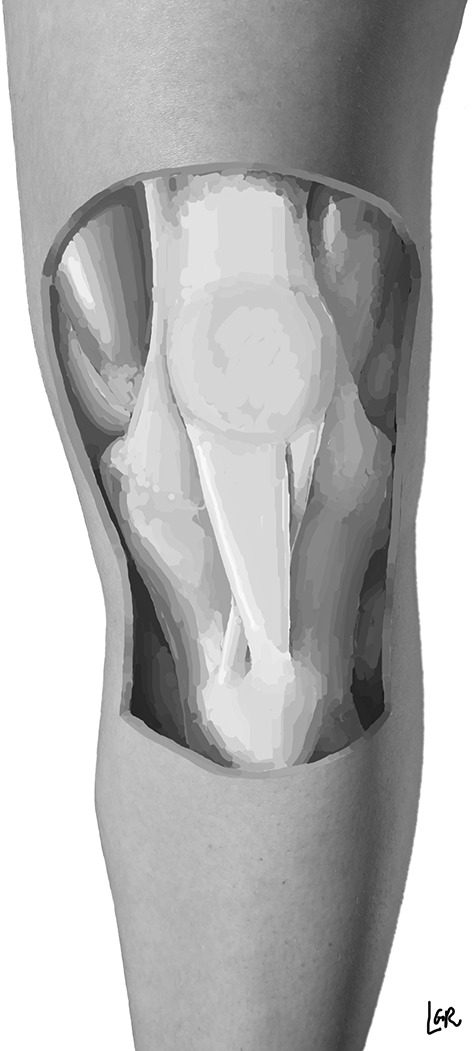
Schematic illustration of the crossed-doubled patellar tendon

In humans, the patellar tendon can be identified in embryos as early as stage 18, whereas in stage 19, the patellar tendon is clearly present in all embryos [6]. The patella itself first becomes visible at the end of stage 19 as cellular condensations and achieves it characteristic shape in stage 20 (Fig. 3).

Variants of anatomic structures can be found throughout the whole body. In the knee region, some variability has been reported, for example, in the quadriceps muscle/tendon: a bilaminar or a doubled vastus medialis muscle or a separate insertion of the vastus medialis passing into the medial condyle of the tibia by a separate tendon is some known variants described in the literature. A fusion between vastus medialis and vastus intermedius has also been reported. In addition, variants of the popliteus musculotendinous unit have been described, such as a bifurcation of the popliteus tendon, a three-bundled popliteus tendon, or cases of an accessory popliteus muscle [4, 10]. In addition, several anatomical variations of the biceps femoris (BF) muscle and tendon have been reported: an absence of the short head of BF, the presence of additional heads that may arise from the sciatic tuberosity, or a case of bilateral tibial insertion of the BF tendon [10]. Concerning the anterior and posterior cruciate ligament, only few anatomical variants exist, which are usually associated with meniscal abnormalities (e.g., discoid meniscus) [4]. Finally, a common bony radiological finding is a bipartite patella, seen in about 2 % of individuals; this is related to a secondary ossification centre that fails to fuse with the main body during patella development [4]. There are no descriptions of (anatomical) variations of the patellar tendon; there are only six reported cases of aplasia [8].

Usually, the PT origins at the anterior aspect of the patella; as a possible anatomic variant, Basso et al. reported six cases on which the tendon fibres originated from the posterior surface of the apex, forming a ridge on the back of the tendon [2]. The situation described here is the first major variant of the patellar tendon described in humans.

In anatomical studies [1, 2], the PT is described as a flat structure deriving from the central portion of the rectus femoris tendon, thin and broad proximally, becoming thick and narrow distally, when the fibre bundles converge as they run towards the tibia [2]. The PT has a mean length between 38 and 49 mm, with a broader and crescent shaped insertion at its insertion into the patella’s apex (31.9 mm) compared to the distal insertion into the tibial tubercle (27.4 mm), which is slightly asymmetrical in relation to the midline [1]. The fascicles are arranged parallel in the sagittal plane, and converge toward their tibial attachment in the frontal plane [2]. As for every tendinous structure, PT shows homogeneous low-signal intensity on all MRI sequences, with the exception of a normally slightly increased signal at the posterior margins of both the origin and insertion of the tendon.

The appearance of the PT after the Roux–Goldthwait procedure [3], a surgical procedure for the treatment of patellar luxation, has similarities with our case: the PT is split vertically, the distal lateral PT-half is separated from its tibial insertion and pulled under the inner medial PT-half and is reinserted at the medial aspect of the tibial tuberosity. In our case, the signal intensity of the two PT tendons was absolutely normal along its whole course, without any signs of recent trauma or scarring. A longitudinally aligned rupture could be ruled out due to the presence of the fatty plane between the tendons, the inclined course, missing oedema, and missing blood decay products. Furthermore, no changes in the adjacent osseous insertion of the PT were found: to exclude any metal induced artefacts, a subsequent MRI gradient-echo sequence (MEDIC, Multi-echo data image combination) was applied. No signs of osseous manipulation or metal abrasion were evident which would have been expected after Roux–Goldthwait surgery. Thus, this anatomical situation turns out to be a genuine congenital developmental variation. It was without any clinical relevance for the patient: the form and the position of the patella were normal, and its stability was not impaired. No sign of degenerative processes was present. The only clinical relevance in evidence consists in concerns regarding removal of a part of these two tendons for crucial ligament repair, supposedly causing an asymmetrical degradation.

As a matter of interest, a very similar arrangement of the anterior region of the knee has been reported for some palaeognathous birds, in particular, the emus, the second-largest living bird in the world [9]. The study from Regnault et al. showed that the emus’ PT, approximately at one-third of its length, splits in a superficial and a deep bundle. The superficial part, similarly to the human PT, crosses over the knee joint and inserts on the cranial part of the bird’s tibia, while the deeper one attaches to the cartilaginous femorotibial menisci. No evidence of any mineralization in the PT of the emus was described, thus assuming that patellar sesamoid was missing [9].

In our opinion, a possible interpretation of our case may be addressed through an understanding of the evolutionary perspective of the knee. In fact, according to comparative anatomical studies, human and avian knees present similarities both in structure and function: a bicondylar distal femoral shape, the presence of menisci, and intra-articular ligaments, as well as collateral ligaments [5]. Thus, this articulation is suggested to have a very old origin, with the Eryops (ancient amphibian appeared on earth approximately 350 million years ago), being considered the oldest common ancestor of reptiles, birds, and mammals [7]. Despite these general similarities, the evolution process leaded to significant differences, being the human the only known species that is biped, but also plantigrade with rather astonishing differences of the inner texture/properties of the PT even compared to our next primate relatives; avian, reptilian, and mammalian knee evolved independently from each other after the common ancient [5]. The resemblance between our cases of a double PT with the PT of the emus may lead us to speculate about the possibility of such a curious and ancestral origin of this anatomical variant.

The patellar tendon is one of the most frequently used grafts in reconstructive knee surgery particularly regarding anterior cruciate ligament reconstruction: it is, thus, of importance knowing the existence of a doubled PT and recognizing this admittedly rare anatomical variant, to avoid misdiagnosis and over-investigation, as well as to provide a more accurate pre-operative planning. In our knowledge, this is the first ever case describing a doubled PT: this rare anatomical variant should be considered especially in the context of patellofemoral stability and surgical procedures.
